# Ferula (*Ferula elaeochytris*) as a phytoestrogen: Use of Ferula in laying hens

**DOI:** 10.3389/fvets.2024.1525101

**Published:** 2025-01-22

**Authors:** Arda Onur Özkök, Gözde Kılınç

**Affiliations:** ^1^Department of Veterinary, Suluova Vocational School, Amasya University, Amasya, Türkiye; ^2^Department of Food Processing, Suluova Vocational School, Amasya University, Amasya, Türkiye

**Keywords:** Ferula, reproductive hormones, oviduct, performance, antioxidant, laying hens

## Abstract

This study aims to examine the effects of Ferula root powder (FRP) on performance, egg quality, egg oxidant/antioxidant levels, some serum hormone/biochemical parameters, and physical properties of the oviduct in laying hens. A total of 72 (8 replicates, 3 hens/subgroup) laying hens (Nick Brown, 30 weeks) were divided into three groups (FRP-0, FRP-1, FRP-2). During the 9-week trial, FRP-0 (control) was fed with a basal diet (16.88% crude protein, 2,725 kcal/kg metabolizable energy). FRP-1 and FRP-2 groups, however, were fed a diet supplemented with 1 g/kg and 2 g/kg FRP, respectively. The results showed that laying performance, serum hormone (estradiol, progesterone) levels, and some internal organ weights were not affected by FRP supplementation. In comparison to the control group, higher yolk height and yolk index were found in the FRP-added groups. The albumen pH was found to have decreased in FRP-2 group. DPPH radical scavenging activity increased in egg yolk. TBARs value decreased in FRP-1 and FRP-2 groups. Serum triglyceride and cholesterol levels decreased in group FRP-2. Moreover, a higher uterus length was found in the FRP-supplemented group. Given the results achieved, it was determined that FRP does not have a significant estrogenic effect. However, FRP can be utilized to prevent lipid oxidation and for its hypocholesterolemic effect.

## 1 Introduction

The use of natural substances that can replace reproductive hormones in farm animals offers significant advantages. There has been interest in studies on this subject in recent years. In particular, isoflavones, phytohormones, carotenoids, and various mineral substances are known to have positive effects on reproductive functions (1). Phytoestrogens are natural, non-steroidal plant components that can mimic the function of estrogen in the body. Their effects can be beneficial or harmful depending on the dose, age, and gender (2).

Steroid hormones including estrogen and progesterone that affect the ovary play an active role in maintaining and terminating pregnancy. Phytosteroids can be used against deterioration in reproductive functions due to some negative factors. They bind to hormone receptors in the reproductive system (3). Therefore, when taken with food and plant-based supplements, phytosteroids can interact with steroid receptors in the target organ and affect the endocrine system (4). It is known that phytoestrogens can either enhance or suppress existing estrogenic effects by mimicking or modifying estrogen receptors in tissues (3).

Ferula belongs to the group of plants having phytoestrogenic effects. Ferula, which belongs to the Apiaceae family, has 170 species known today (5). Ferula plant contains ferutinin, which has hypoglycemic and estrogenic properties. This substance has an agonistic effect on estrogen receptors. For this reason, it was stated that this material is used in some countries to eliminate various infertility problems (6). Direct application of ferutinin can have estrogenic or antiestrogenic effects depending on the estrogen activity status (7). It is known that the compounds contained in the root, leaf, and rhizome of Ferula have different pharmaceutical effects. Even though ferulenol, which is included in the composition of Ferula, has a lethal effect when taken at high doses, it has an effect that can tolerate estrogen deficiency when used at low doses (8). The composition of Ferula species includes coumarin and coumarin esters, monoterpene coumarins, prenylated coumarins, various sulfur-containing compounds, sesquiterpenes, sesquiterpene lactones, carbohydrates, flavonoids, and phytoestrogens that have estrogenic effects *in vivo* (9). In addition, ferula species also contain cancer-preventing components such as ferprenin, ferrutinin, farnesifenol A, farnesivenol B, daucan esters, galbanic acid, sinciangenorin C, sinciangenorin E, umbelliprene and aurapren. In addition, terpene biosynthesis in ferula roots is much higher than in other parts of the plant (10). Terpenoids are known to have anti-inflammatory, antibacterial, and antiviral effects, reduce the incidence of cardiovascular diseases, and regulate sugar levels in blood (11). The terpenoid structure found in Ferula species has some components that stimulate estrogenic activity. Among the approximately 200 types of estrogenic plants in the world, 60 of them belong to the Ferula species (12). *Ferula elaeochytris* is a perennial plant, which is native to Türkiye. Also known as çakşır (a type of sage), it is commonly utilized for its aphrodisiac, anti-inflammatory, anti-diabetic, and antioxidant features (5). *F. elaeochytris* was reported to contain 25.9% khusinol, known for its anti-inflammatory effect, 13.9% ferutinine, an estrogenic component, and beta-ionone, as well as 22% beta-ionone, which has antiproliferative and antioxidative effects (13).

Reviewing the literature, it was determined that there are many studies examining the effect of Ferula on performance and egg quality in poultry. However, the number of studies on the effects of ferula, known for its phytoestrogenic properties, on reproductive hormones, follicles, and oviducts in laying hens is limited. This study aims to examine the effects of Ferula (*F. elaeochytris*) root powder in diets of laying hens on laying performance, egg quality traits, oxidant/antioxidant levels of eggs, the concentrations of some blood serum parameters and serum reproductive hormones, internal organ weights, follicles, and some parts of the oviduct.

## 2 Materials and methods

### 2.1 Ethical approval

This study was approved by the Animal Ethics Committee of Ondokuz Mayıs University, Türkiye (2023/95).

### 2.2 Preparation of FRP and chemical analysis

Ferula was collected with its roots from the Yayladağı district of Hatay province. The species of the plant was identified as *Ferula elaeochytris* by Prof. Dr. Cengiz YILDIRIM (Amasya University, Faculty of Education, Türkiye). Ferula roots were separated and rinsed. Then, the samples were dried in an drying oven (Gemo DT107) at 45°C and powdered using a laboratory blender (Waring Laboratory Blender, United States).

Proximate composition of FRP was determined by using the process introduced by the Association of Official Analytical Chemists, AOAC (14).

Volatile oil constituents were analyzed by using gas chromatography/mass spectrometry (GC–MS; Agilent:6890 MS:5973, New Jersey, USA). Component mass spectra were determined by comparing them with the current libraries (Flavor2, W8N05ST, and HPCH1607). Volatile compounds with a confidence level lower than 50% were not considered during the evaluation.

### 2.3 Experimental plan, management, and diets

The animal experiments within the scope of this study were conducted in the Experimental Poultry Unit of Suluova Vocational School of Amasya University, Türkiye. A total of seventy-two (8 replicates, 3 hens/subgroup) 30-week-old Nick Brown commercial hens were randomly allocated to 4-storey cages as three groups (FRP-0, FRP-1, and FRP-2). The number of replicates in each group was calculated by using G*Power (version, 3.1.9.4) package software. The homogeneity of variances was tested for initial body weight (*p* > 0.05). The experimental design is given in Table 1.

**Table 1:** Experimental design.

Groups	Replicates	Hens in subgroups	Experimental diets
FRP-0	8	3	Basal (control) diet
FRP-1	8	3	1 kg basal diet +1 g FRP
FRP-2	8	3	1 kg basal diet +2 g FRP

FRP, Ferula root powder.

During the experiment, which took 8 weeks after a 1-week adaptation period, feed/water was given *ad-libitum*, and a photoperiod 16 h of light and 8 h of dark was performed. The temperature in the experiment unit was controlled by using a digital thermometer (data logger, testo 174 T) for 24 h and kept at 22–24°C by making use of ventilation.

FRP-0 (control) was fed a basal diet (Table 2). However, the diet given to FRP-1 and FRP-2 groups was supplemented with 1 g/kg and 2 g/kg FRP, respectively.

**Table 2:** The composition and nutrient levels of basal feed.

Items	kg/ton
Maize	475.00
Soybean meal (46% CP)	41.44
Full fat soybean (34% CP)	150.00
Wheat	143.86
Sunflower seed meal (34% CP)	86.29
Sunflower oil	5.10
Limestone	88.00
Dicalcium phosphate	3.79
Salt	2.50
DL-Methionine	0.87
Lisin sulphate	0.65
Premix*	2.50
Chemical composition (analyzed) %	
Dry matter	89.08
Crude protein	16.88
Ether extract	5.49
Crude ash	11.92
Calcium	3.60
Metabolizable energy (kcal/kg)**2275	

^*^Supplied the following per 2.5 kg of diet: 10.000.000 IU vitamin A, 3.000.000 IU vitamin D3, 25.000 mg vitamin E, 4000 mg vitamin K3, 2.000 mg vitamin B1, 5.000 mg vitamin B2, 40.000 mg niacin, 12.000 mg calcium D pantothenate, 4.000 mg vitamin B6, 20 mg vitamin B12, 1.000 mg folic acid, 60 mg biotin, 120.000 Mn, 40.000 mg Fe, 70.000 Zn, 7.000 mg Cu, 500 mg Se, 2.000 mg canthaxanthin, and 1.200.000 phytase.

^**^The metabolizable energy level was calculated as stated by Carpenter and Clegg (50).

The composition and nutrient levels of FRP are presented in Table 3.

**Table 3:** Some nutrient composition of Ferula (*F. elaeochytris*) root.

Items	%
Dry matter	93.4
Crude protein	4.3
Ether extract	4.0
Crude fiber	16.0
Crude ash	9.0

### 2.4 Sample collection

To determine performance parameters (egg weight, feed conversion ratio, egg production, feed intake, egg mass), feed was weighed and administered daily, and eggs were also counted and recorded daily. Performance parameters were determined bi-weekly. Egg quality (egg width and length, shape index, yolk diameter, yolk height, albumen height, albumen index, yolk index, Haugh unit, shell thickness, shell weight, egg yolk’s color L, a, b values, and albumen pH) was analyzed on 20 eggs collected from each group after the trial was completed. TBARs values were determined for 10 eggs collected at the end of the experiment. Moreover, DPPH radical scavenging analysis was conducted on 20 eggs. At the end of the experiment, approximately 4 mL of blood was collected from seven hens through intravenous puncture into yellow-capped vacuum tubes to assess certain serum biochemistry parameters (aspartate transaminase, alanine aminotransferase, alkaline phosphatase, total protein, triglycerides, and total cholesterol) and reproductive hormone concentrations (estradiol and progesterone). These samples were then centrifuged (Hermle Z206A) at 3000 rpm for 10 min to separate the serum, and the serum samples were kept at −21°C until analysis.

After the completion of the experiment, 7 hens from each group were killed by cutting the jugular vein. Then, some of their internal organs (liver, spleen, gizzard, and heart) were weighed. The oviducts and follicles of the same animals were removed, and total follicle weight, graff follicle weight, total oviduct weight, uterus width, uterus length, and graff follicle diameter were determined.

### 2.5 Traits measured

Egg weights were determined by using a scale with 0.01 g precision (KERN PFB). Egg production (15), feed conversion ratio (16), egg mass (17), and feed consumption (18) were calculated by making use of the following formulae.


$$\mathrm{Feed}\;\mathrm{consumption}\;\left(\begin{array}{l} \mathrm{kg}\;\mathrm{feed}/\\ \mathrm{kg}\;\mathrm{egg} \end{array}\right)=\frac{\begin{array}{l} \mathrm{Total}\;\mathrm{feed}\;\mathrm{given}-\\ \mathrm{remaining}\;\mathrm{feed}\; \end{array}}{\mathrm{Day}\;X\;\mathrm{Hens}}\;\times 100$$

$$\mathrm{Feed}\;\mathrm{conversion}\;\mathrm{ratio}\;\left(\begin{array}{l} \mathrm{kg}\;\mathrm{feed}/\\ \mathrm{kg}\;\mathrm{egg} \end{array}\right)=\frac{\begin{array}{l} \mathrm{Avarege}\;\mathrm{feed}\;\\ \mathrm{consumption}\; \end{array}}{\begin{array}{l} \mathrm{Avarege}\;\mathrm{eggs}\;\\ \mathrm{weight} \end{array}}\;\times 100$$

$$\mathrm{Egg}\;\mathrm{production}\;\left(\%\right)=\frac{\mathrm{Total}\;\mathrm{number}\;\mathrm{of}\;\mathrm{eggs}\;}{\mathrm{Day}\times \mathrm{Hens}}\times 100$$

$$\mathrm{Egg}\;\mathrm{mass}\;\left(g/\mathrm{hen}/\mathrm{day}\right)=\mathrm{Avarege}\;\mathrm{egg}\;\mathrm{weight}\times \mathrm{Egg}\;\mathrm{production}$$

Egg width, egg length, yolk diameter, albumen length, and albumen width values were determined by using a digital caliper (1108–150, Insize, China). Albumen height and yolk height values were determined by using a tripod micrometer (Mitutoyo, 0.01–20 mm; Kawasaki, Japan). Considering these values, the shape index (19), albumen index (20), yolk index (21), and Haugh unit (22) values were obtained using following formulae.


$$\mathrm{Shape}\;\mathrm{index}\;\left(\%\right)=\frac{\mathrm{Egg}\;\mathrm{width}\;\left(\mathrm{mm}\right)}{\mathrm{Egg}\;\mathrm{lenght}\;\left(\mathrm{mm}\right)}\times 100$$

$$\mathrm{Albu}men\;\mathrm{index}\;\left(\%\right)=\frac{\mathrm{Albumen height}\;\left(\mathrm{mm}\right)}{\left(\begin{array}{l} \mathrm{Albumen}\;\mathrm{width}+\\ \mathrm{Albumen}\;\mathrm{lenght} \end{array}\right)/2\;\left(\mathrm{mm}\right)}\times 100$$

$$\mathrm{Yolk}\;\mathrm{index}\;\left(\%\right)=\frac{\mathrm{Yolk}\;\mathrm{height}\;\left(\mathrm{mm}\right)}{\mathrm{Yolk}\;\mathrm{lenght}\;\left(\mathrm{mm}\right)}\times 100$$

$$\mathrm{Haugh}\;\mathrm{unit}\;\left(\%\right)=100\times \log\;\left(H+7.57-1.7W^{0.37}\right)$$

H = Albumen height, W = Egg weight.

Eggshells were weighed using a digital scale (0.001 g precision, RADWAG, AS 220.R2 Plus). Eggshell thickness was measured by using a micrometer (Mitutoya, No. 1044 N, 0.01–5 mm; Kawasaki, Japan) on three different parts of shells (upper, middle, and lower).

L, a, and b values of yolks were determined by using a colorimeter (PCE-CSM 4). L, a, and b values refer to lightness, redness, and yellowness, respectively.

In order to measure albumen pH, the albumen was separated and homogenized (Ultra turrax; CAT, 33.000 rpm) for 20 s. Then, pH levels of albumen samples were measured by using a pH meter (OHAUS, Aquasearcher AB41PH, NJ, USA) that was calibrated (23).

The malondialdehyde (MDA) concentrations were determined as an index of lipid peroxidation and thiobarbituric acid reactive substances (TBARs) values were calculated by making use of the following formulae as μmol MDA/kg egg.

$\mathrm{TBARS}=\left[\left(\begin{array}{l} \mathrm{absorbance}/\\ k\left(0.06\right)\times 2/1000 \end{array}\right)\times 6.8\right]\times 1000/\mathrm{sample weight}$

*k* = Value obtained from the standard curve.

Yolk MDA values were measured by following the method developed by Tarladgis et al. (24) and modified by Lemon (25).

Two grams of yolk were weighed in a 50 mL beaker and 12 mL trichloro-acetic acid (TCA) solution prepared using EDTA (1%), propyl gallate (1%), and TCA (7.5%) was added. The mixtures were homogenized (Ultra turrax; CAT, 33.000 rpm) for 15 s. TCA-yolk mixtures were filtrated through Whatmann (No.1) filter paper in a 25 mL beaker, 3 mL of filtered samples were taken in a glass tube, and thiobarbituric acid (0.02 M) was added. The tubes were kept at 100°C in a water bath (Wise Bath) for 40 min. Then, the absorbance values were determined by making use of a spectrophotometer (Genesys 10S UV–VIS, Therma Scientific) at 530 nm wavelength.

DPPH radical scavenging activity values were determined by following the methods of Farıvar (26). Two grams of yolk were weighed in a 45 mL falcon conical tubes, and 25 mL methanol was added. The tubes were then placed in an ultrasonic bath (CALISKAN Ultrasonic Cleaner) for 25 min. Methanol-yolk mixtures were filtrated using Whatmann (No.1) filter paper in a 25 mL beaker. Then, 100 μL of filtrated samples were taken in a 15 mL glass conical tube, and 2.9 mL DPPH solution (0.0025 g DPPH +100 mL methanol) was added. The tubes were vortexed for 20 s. and then incubated for 60 min. Subsequently, the absorbance values were determined by making use of a spectrophotometer (Genesys 10S UV–VIS, Therma Scientific) at 517 nm wavelength. DPPH radical scavenging activity values were evaluated by making use of the following formula (27) as %.

$\mathrm{DPPH radical scavenging activity}\;\left(\%\right)=\left(A0-A1\right)/A0\times 100$ .

A0: Absorbance of the control (free yolk-sample), A1: Absorbance of the samples.

Serum aspartate aminotransferase (AST, ottoBC127), alkaline phosphatase (ALP, ottoBC124), alanine aminotransferase (ALT, ottoBC128), triglyceride (ottoBC155), total protein (ottoBC154), and total cholesterol (ottoBC135) concentrations were measured by using an auto-analyzer (Mindray-BS400) with commercial test kits (Otto Scientific, Türkiye).

Serum progesterone (ng/ml) and Estradiol (pg/ml) hormone levels were determined by using electrochemiluminescence immunoassays - ECLIA (Roche, e601) with commercial kits (Roche).

The weights of liver, spleen, gizzard, and heart were determined using a digital scale (0.001 g precision, RADWAG, AS 220.R2 Plus).

The oviducts and follicles of hens cutting were removed. Then, the measurements were performed following the method introduced by Patil et al. (28). Total follicle, graff follicle, and total oviduct weights were measured using a digital scale (0.01 g precision, KERN PFB). Additionally, uterus width, uterus length, and graff follicle diameter were measured using a digital caliper (Insize, 1,108–150, China).

### 2.6 Statistical analysis

The SPSS 21.0 package program was used in the statistical analysis process and One-way ANOVA procedure in determining the difference between groups. Duncan’s *post hoc* test was utilized in comparing the differences among the means of groups. Effects of dietary FRP levels (1 g/kg and 2 g/kg) were determined using polynomial contrasts. *p* ≤ 0.05 values were considered statistically significant (29). In addition, numbers of replicates for hens in this study were calculated using the G*Power (version 3.1.9.4) package software (30).

## 3 Results

### 3.1 Characteristics of FRP

The results of the GC/MS analysis of volatile oil isolated from roots of the *F. elaeochytris* are given in Table 4. Given the results achieved, there are a total of 52 components in this volatile oil. Major compounds are carvacrol (28.6%), cadalene (9%), and E-nerolidol (4.8%), respectively.

**Table 4:** Volatile oil profile of Ferula (*F. elaeochytris*) root.

Volatile oils	g/100 g	Volatile oils	g/100 g
m-Tolualdehyde	2.0	Thujopsan-2-alpha-ol	0.4
p-Tolualdehyde	0.8	1,4-Methanobenzocyclodecene	1.0
Ethanone, 1-(2-hydroxphenyl)	0.4	1,4-Methanoazulen-7-ol,decahydro-	0.7
Carvone	0.4	Benzene	2.1
Phenol, 4-(1-methylethyl)	0.5	trans-beta-Guaine	0.2
Cyclohexanone, 2-methyl-5-(1-methylethenyl)-,(25-cis)	0.4	Neo-Intermedeol	0.3
p-Cymen-7-ol	1.1	Cyclohexane	1.5
Benzoic acid,	0.4	Oplopenone	3.5
Carvacrol	28.6	Viridiflorol	1.0
1-10-Decanediol	1.5	Cadalene	9.0
β-Citronellene	0.5	Helifolen-12-AL D	1.1
Geranylaceton (isomer 2)	4.3	1,2-Dimethyl-3-ethylbenzene	0.3
ar-Curcumene	0.8	ar-Turmerol	0.7
alpha-trans-Bergamotene	0.9	Helifolen-12-AL B	0.4
Cuparene	2.3	E-Sesquilavandulol	0.6
β-Bisabolene	1.8	E-Nuciferol	0.9
Hexahydro-1-methyl-2H-azepine-2-thione	2.3	Himachalol	0.2
Nerolidoloxide	1.2	neo-allo-Ocimene	2.0
Hexanedioic acid, 3-methyl-, bis (1-methyl propyl) ester	1.3	Furanmethanol	3.6
Bisabolol oxide A	0.4	Z-Lanceol	1.0
Bicylo (2.2.1.) heptane,2,2-dimethyl-3-methylene	1.4	Z-alpha-trans-Bergamotol	2.6
E-Nerolidol	4.8	alpha-Curcumen	0.6
2,6-Octadien-1-ol,3,7-dimethyl-acetate	0.5	Diisobutyl phthalate	0.8
Benzene, 1-(1.5-dimethyl-4-hexenyl)-4-methyl-	0.3	β-Curcumene	0.2
3,5,9-undecatrien-2-one, 6,10-dimethyl-,(E,E)	0.6	Z,E-Farnesol	2.1
Cyclohexene, 1,3,3-trimethyl-6-methylene	2.5	Ethanone, 1-(1,4-dimethyl-3-cychexen-1-YL)	1.2

### 3.2 Productive performance

The impacts of supplementing FRP (0, 1, and 2 g/kg) in diet on productive performance are presented in Table 5 (1–28 days), 6 (28–56 days), and 7 (1–56 days). The results indicate that adding FRP to the diets of laying hens did not affect egg weight, feed conversion ratio, feed consumption, egg production, and egg mass (Tables 5-7).

**Table 5:** Effects of dietary supplementation of Ferula (*F. elaeochytris*) root powder on performance parameters (1–28 days).

Items	Ferula root powder (g/kg)			SEM	*p*-values	
	0	1	2		Combined	Linear
Egg weight, g	63.89	65.05	64.10	0.333	0.319	0.797
Feed conversion ratio, kg feed/kg egg	1.86	1.85	1.86	0.016	0.914	0.988
Feed consumption, g	118.93	120.10	119.40	0.804	0.841	0.816
Egg production, %	86.50	93.19	87.17	1.587	0.166	0.862
Egg mass, g/hen/day	55.37	60.63	55.85	1.106	0.098	0.854

*n = 16.*

SEM, Standard error of mean.

**Table 6:** Effects of dietary supplementation of Ferula (*F. elaeochytris*) root powder on performance parameters (28–56 days).

Items	Ferula root powder (g/kg)			SEM	*p*-values	
	0	1	2		Combined	Linear
Egg weight, g	64.04	64.68	63.12	0.377	0.245	0.323
Feed conversion ratio, kg feed/kg egg	1.75	1.75	1.82	0.014	0.053	0.036
Feed consumption, g	111.95	113.14	114.99	0.772	0.274	0.112
Egg production, %	93.45	93.75	92.26	1.389	0.902	0.733
Egg mass, g/hen/day	59.93	60.77	58.24	1.039	0.609	0.514

*n = 16.*

SEM, Standard error of mean.

**Table 7:** Effects of dietary supplementation of Ferula (*F. elaeochytris*) root powder on performance parameters (1–56 days).

Items	Ferula root powder (g/kg)			SEM	*p*-values	
	0	1	2		Combined	Linear
Egg weight, g	63.96	64.87	63.91	0.251	0.109	0.562
Feed conversion ratio, kg feed/kg egg	1.81	1.80	1.84	0.011	0.263	0.208
Feed consumption, g	115.44	116.62	117.20	0.637	0.523	0.265
Egg production, %	89.97	93.47	89.71	1.071	0.281	0.921
Egg mass, g/hen/day	57.65	60.70	57.05	0.765	0.112	0.745

*n = 32.*

SEM, Standard error of mean.

### 3.3 Egg quality

The impacts of FRP (0, 1, and 2 g/kg) in the diets of laying hens on egg quality traits are summarized in Table 8. Higher yolk height (*p* = 0.001) and yolk index (*p* < 0.05) values were achieved in the FRP-added groups when compared to the control group. The albumen pH (*p* < 0.05) was determined to be at a lower level in FRP-2 group. Other egg quality parameters in Table 8 were not affected by FRP supplementation.

**Table 8:** Effects of dietary supplementation of Ferula (*F. elaeochytris*) root powder on egg quality parameters.

Items	Ferula root powder (g/kg)			SEM	*p*-values	
	0	1	2		Combined	Linear
Egg width, mm	44.17	44.82	44.82	0.210	0.355	0.213
Egg length, mm	56.58	56.96	56.33	0.198	0.427	0.610
Shape index, %	78.08	78.73	79.58	0.372	0.260	0.103
Yolk diameter, mm	43.32	43.49	43.23	0.142	0.761	0.805
Yolk height, mm	18.29^b^	18.84^a^	18.82^a^	0.072	0.001	0.001
Albumen height, mm	6.46	6.75	6.65	0.084	0.365	0.347
Yolk index, %	42.25^b^	43.35^a^	43.59^a^	0.232	0.038	0.016
Albumen index, %	7.930	8.472	8.520	0.143	0.174	0.092
Haugh unit, %	79.19	80.66	80.57	0.612	0.556	0.366
Yolk color L	52.62	55.63	56.11	0.982	0.296	0.152
Yolk color a	20.70	20.56	20.49	0.166	0.875	0.613
Yolk color b	47.69	48.13	47.99	0.414	0.909	0.768
Albumen pH	7.883^a^	7.892^a^	7.818^b^	0.041	0.042	0.126
Shell weight, g	6.24	6.39	6.37	0.075	0.668	0.468
Shell thickness, μm	0.353	0.361	0.361	0.003	0.523	0.316

*n = 20.*

SEM, Standard error of mean.

L, lightness, a, redness, and b, yellowness.

Different superscript letters (a, b) within the same row mean significant differences between groups (*p* < 0.05).

### 3.4 Egg oxidant and antioxidant parameters

The impacts of FRP (0, 1, and 2 g/kg) in the diets of laying hens on oxidant (TBARs) and antioxidant (DPPH radical scavenging activity) levels are presented in Table 9. FRP-3 (8.731%) was found to have an increase (*p* < 0.05) in DPPH radical scavenging activity values when compared to FRP-0 (control) and FRP-1. TBARs levels in yolk were found to have decreased (*p* < 0.05) in FRP-1 (1.238 μmol MDA/kg egg) and FRP-2 (1.034 μmol MDA/kg egg) groups in comparison to the FRP-0 group. Given the results of the polynomial analysis, there was a significant linear effect for both TBARs (*p* = 0.001) and DPPH (*p* = 0.001) levels. A linear increase was observed in DPPH level and a linear decrease in TBARs level with increasing dose of FRP.

**Table 9:** Effects of dietary supplementation of Ferula (*F. elaeochytris*) root powder on oxidative stability of eggs.

Items	Ferula root powder (g/kg)			SEM	*p*-values	
	0	1	2		Combined	Linear
TBARs (μmol MDA/kg egg)	1.822^a^	1.238^b^	1.034^b^	0.102	0.002	0.001
DPPH (%)	7.991^b^	8.100^b^	8.731^a^	0.096	0.002	0.001

*n* = 10 (TBARs), *n* = 20 (DPPH).

SEM, Standard error of mean.

TBARs, Thiobarbituric acid reactive substances; MDA, Malondialdehyde; DPPH, 2,2-diphenyl-1-picrylhydrazyl.

Different superscript letters (a, b) within the same row mean significant differences between groups (*p* < 0.05).

### 3.5 Serum biochemical parameters

Table 10 presents the results obtained for some serum biochemical parameters. Except for serum triglyceride (*p* < 0.05) and cholesterol (*p* < 0.05) levels, other serum biochemical parameters (AST, ALP, ALT, and total protein), were not affected by FRP supplement. Serum triglyceride (947.10 mg/dL) and cholesterol (59.49 mg/dL) levels were found to have decreased in FRP-2 group. The linear effect was significant in terms of triglyceride and total cholesterol concentrations in serum.

**Table 10:** Effects of dietary supplementation of Ferula (*F. elaeochytris*) root powder on some serum biochemical parameters.

Items	Ferula root powder (g/kg)			SEM	*p*-values	
	0	1	2		Combined	Linear
AST, U/L	151.40	158.86	156.54	2.933	0.593	0.494
ALT, U/L	24.70	25.52	28.42	4.135	0.940	0.742
ALP, U/L	432.60	466.73	470.83	8.374	0.121	0.062
Total protein, g/dL	10.26	11.68	11.53	0.315	0.128	0.097
Triglyceride, mg/dL	1545.32^a^	1136.99^ab^	947.10^b^	98.69	0.031	0.011
Total cholesterol, mg/dL	64.92^a^	68.63^a^	59.49^b^	1.282	0.007	0.044

*n = 7.*

SEM, Standard error of mean.

ST, Aspartate aminotransferase; ALT, Alanine aminotransferase; ALP, Alkaline phosphatase.

Different superscript letters (a, b) within the same row mean significant differences between groups (*p* < 0.05).

### 3.6 Serum reproductive hormones

The mean concentrations of some serum reproductive hormones are given in Table 11. Considering the results presented, it can be seen that serum estradiol and progesterone concentrations were not affected by FRP supplementation (*p* > 0.05).

**Table 11:** Effects of dietary supplementation of Ferula (*F. elaeochytris*) root powder on some serum hormone parameters.

Items	Ferula root powder (g/kg)			SEM	*p*-values	
	0	1	2		Combined	Linear
Estradiol (E2), pg./mL	257.86	273.81	279.73	12.564	0.781	0.503
Progesterone, ng/mL	0.224	0.171	0.287	0.024	0.138	0.269

*n = 7.*

SEM, Standard error of mean.

### 3.7 Internal organ weight

The effects of FRP (0, 1, and 2 g/kg) supplement on some internal organ weights are summarized in Table 12. It can be seen that liver, spleen, gizzard, and heart weights were not affected by FRP supplementation.

**Table 12:** Effects of dietary supplementation of Ferula (*F. elaeochytris*) root powder on some internal organ weight.

Items	Ferula root powder (g/kg)			SEM	*p*-values	
	0	1	2		Combined	Linear
Liver weight, g	32.84	33.13	33.78	0.395	0.629	0.353
Spleen weight, g	1.52	1.37	1.47	0.054	0.555	0.700
Gizzard weight, g	26.87	26.10	27.38	0.303	0.229	0.492
Heart weight, g	6.25	5.91	6.09	0.132	0.607	0.643

*n = 7.*

SEM, Standard error of mean.

### 3.8 The follicles and physical properties of oviduct

The effects of FRP (0, 1, and 2 g/kg) supplement on the follicles and physical properties of the oviduct are presented in Table 13. Total follicle weight, graff follicle weight, total oviduct weight, uterus width, and graff follicle diameter were found to have not been affected (*p* > 0.05) by FRP supplement but there was an effect on uterus length by FRP supplementation (*p* < 0.05). A significant increase was found in uterus length in FRP-1 and FRP-2 groups (*p* < 0.05).

**Table 13:** Effects of dietary supplementation of Ferula (*F. elaeochytris*) root powder on follicles and some parts of oviduct.

Items	Ferula root powder (g/kg)			SEM	*p*-values	
	0	1	2		Combined	Linear
Total follicle weight, g	36.86	41.62	41.41	1.276	0.235	0.151
Graff follicle weight, g	11.81	12.40	13.16	0.265	0.112	0.039
Total oviduct weight, g	60.66	64.49	62.48	1.160	0.441	0.541
Uterus width, mm	51.56	51.06	54.77	0.866	0.168	0.130
Uterus length, mm	81.41^b^	93.47^a^	92.15^a^	1.715	0.002	0.003
Graff follicle diameter, mm	30.81	33.04	32.82	0.481	0.108	0.083

*n = 7.*

SEM, Standard error of mean.

Different superscript letters (a, b) within the same row mean significant differences between groups (*p* < 0.05).

## 4 Discussion

Given the results achieved, 52 components were identified in *F. elaeochytris* root powder (Table 4). The top three major compounds are carvacrol (28.6%), cadalene (9%), and E-nerolidol (4.8%), respectively. Duru and Şahin (31) stated in their study that the three major components in the female *F. elaochytris* root were alpha-pinene (76.74%), 2-beta pinene (6.49%), and 7-methyl-1,3,5 cycloheptatriene (2.76%), whereas the three major components in the male *Ferula elaochytris* root were alpha-pinene (87.72%), 2-beta pinene (4.03%), and cyclohexene, 1-methyl-4- 1-methyl (1.99%). Başer et al. (32) determined that the three major components of *Ferula elaeochytris* root powder were nonane (27.1%), *α*-pinene (12.7%), and germacrene B (10.3%), respectively. In the present study, it was found that FRP did not significantly affect the performance parameters. In a previous study, Duru and Şahin (31) reported that dietary male and female Ferula (*F. elaeochytris*) addition at different levels (0, 5, and 10 g/kg) did not affect performance parameters in laying hens. From this aspect, the present study is similar to the study carried out by Duru and Şahin (31). In another study (33), uncoated/coated Ferula (*F. elaeochytris*) root powder (0, 5, and 10 g/kg) added to broiler chicks and layer chicken diets did not affect the production performance of laying hens and growth performance of broiler chicks. Similarly, Filik (34) reported that *F. elaeochytris* root powders (0, 2, 4, and 8 g/kg) did not affect performance of layer hens. In another study, it was determined that *F. elaeochytris* (0, 10, and 20 g/kg) root powder did not affect the performance of laying hens (35). It can be seen that the results reported in these studies and those achieved in the present study are also parallel in terms of performance parameters. Canogullari et al. (36) also investigated the effects of Ferula (*F. elaeochytris*; 0, 5, and 10 g/kg) root powder on growth performance and reproductive performance in their studies on Japanese quails. In this study, it was found that the supplementation of Ferula to the grower diet in the first stage of the trial (growth period) did not affect FCR and feed consumption. In the second stage of the trial (reproductive period), it was determined that live weight, sexual maturity, first egg weight, mean egg weight, and egg production were not affected by Ferula supplement in the diet. Olmez et al. (37) reported that root powder (%0, 0.1, and 0.2) of another species of Ferula (*F. communis* L.) did not affect feed consumption, egg weight, and FCR in Japanese quails. In addition, in a study (38) carried out on Gold Fish (*Carassius auratus* L. 1758), it was reported that the effect of Ferula (*F. elaeochytris*; 1, 5, and 10 g/kg) on live weight gain, growth length, FCR, and condition factor was not significant. There are also studies that are contrary to the present study from the aspect of performance parameters. In a study carried out by Hao et al. (39) on aged layer hens, the effects of Ferula (0, 50, 100, and 200 mg/kg) dietary supplementation on productive and reproductive performance were investigated. In their study, it was reported that 100 mg/kg dietary supplementation of Ferula increased laying rate, and egg weight and improved FCR. Olmez et al. (37) stated that 0.1% dietary Ferula (*F. communis* L.) in Japanese quail diets reduced egg production and 0.2% increased egg production. In another study, Tayeb (40) determined that different levels (10, 15, and 20%) of Ferula (*Ferula communis* L.) addition in the diets of Japanese quail increased live weight and live weight gain and decreased mortality. In a study on broiler chickens (41), it was stated that the addition of *Ferula badrakema* root powder (0.75 and 1.5%) to feed containing 0.15% saccharin reduced feed consumption. In the present study, higher yolk height (*p* = 0.001) and yolk index (*p* < 0.05) results were achieved in the FRP-added groups when compared to the control group. The albumen pH (*p* < 0.05) value was found to have decreased in FRP-2 group. Other egg quality parameters were not affected by supplemental FRP. The decrease in albumen pH of the egg indicates an improvement in shelf life. This outcome is thought to be due to the phytochemicals contained in *F. elaeochytris.* However, this was not reflected in the Haugh unit and albumen index parameters. Some studies (31, 33, 34) in laying hens revealed that *F. elaeochytris* did not significantly affect egg quality traits. In some studies on turkeys, laying hens, and quails, it was observed that Ferula had statistically significant effects on yolk color (37), yolk weight (42), shell percentage (37), albumen index (37), eggshell weight (35), albumen width (35), and shape index (42). In their study on Japanese quails, Olmez et al. (37) found that dietary 0.1% *F. communis* L. root powder improved the yellow color and albumen index. In the same study, 0.1% Ferula supplement decreased eggshell percentage, whereas 0.2% supplement concentration yielded an increase in eggshell percentage. In the present study, yolk TBARs levels decreased in hens receiving supplemental ferula, whereas DPPH radical scavenging activity increased in comparison to the control group. There was a linear increase in DPPH level and a linear decrease in TBARs level with increasing dose of FRP. Thus, these results indicate an improvement in the shelf life of the egg. This situation is thought to be related to the antioxidant effect of the bioactive substances contained in *F. elaeochytris*. Many Ferula species are known to have an important regulatory effect against oxidative stress and exhibit a strong antioxidative effect by reducing the level of malondialdahyde, reactive oxygen species (ROS), nitric oxide, while increasing the activity of superoxide dismutase, catalase, and glutathione peroxidase (43). Contrary to the present study, a study carried out by Duru (33) revealed that coated or uncoated *F. elaeochytris* root powder (5 and 10 g/kg) did not affect the TBA (3-day and 21-day storage) level and therefore did not improve the shelf life of chicken meat. Given the results achieved in the present study, Ferula supplementation to diets of laying hens did not affect serum enzyme activities (ALT, ALP, and AST) and total protein levels statistically significantly, but decreased serum triglyceride and total cholesterol concentrations. The lowest total cholesterol and triglyceride levels were found in the FRP-2 group. Therefore, it is thought that the reduction of serum triglyceride and total cholesterol with *F. elaeochytris* is due to the hypococholestrolemic effects of its active ingredients. In a previous study, Duru (33) reported that 5 and 10 g/kg *F. elaeochytris*, coated or uncoated, did not affect concentrations of plasma metabolites (plasma glucose, calcium, total protein, total cholesterol, and triglyceride) in broiler chickens. In the same study, Duru (33) concluded that Ferula did not affect egg cholesterol levels in experiments on laying hens. In contrast to the present study, Filik (34) reported that 8 g/kg *F. elaeochytris* supplement in laying hen diets increased plasma cholesterol levels. In the same study, Filik (34) determined that plasma calcium levels were not affected by dietary Ferula supplement and that 4 g/kg Ferula addition decreased egg cholesterol (especially for the 3^rd^, 4^th^, and 5^th^ weeks) levels. Similar to the present study, Khajavi et al. (41) stated that the addition of Ferula (*F. badrakema*) root powder (0.75 and 1.5%) at different concentrations reduced blood cholesterol and triglyceride levels of broiler chickens. Khajavi et al. (41) also reported that ferula increased uric acid, albumin, and phosphorus concentrations in blood and that dietary Ferula increased total antibody titers and IgG levels in broiler chickens at the age of 35 days. In another study, Tayeb (40) reported that *F. communis* L. reduced serum albumin and glucose levels in Japanese quails. In a study on koi carp (*Cyprinus carpio* koi), Safari et al. (44) investigated the effects of different concentrations (0, 5, 10, 15, 20, and 25 g/kg) of *F. asafoetida* (flower, leaf, and root mixture) powder on blood parameters. Ferula supplement in the diets of koi carp increased WBCs, RBCs, MCV, MCH, hematocrit, and hemoglobin concentrations in a study carried out by Safari et al. (44). Moreover, Safari et al. (44), also reported an increase in serum total protein level and total immunoglobulin activity. Internal organ (liver, spleen, gizzard, and heart) weights were not affected by FRP supplementation in the diet of laying hens in the present study. In a study carried out by Khajavi et al. (41), it was found that *F. badrakema* root powder (0, 0.75, and 1.5%) in broiler chicken feeds did not significantly affect carcass characteristics (gizzard, heart, liver, small intestine, abdominal fat, thigs, and edible carcass). The serum estradiol and progesterone concentrations were not affected by FRP supplementation in the diet of laying hens in the present study (*p* > 0.05). Total follicle weight, graff follicle weight, total oviduct weight, uterus width, and graff follicle diameter were not affected (*p* > 0.05) by FRP supplementation. However, uterus length significantly increased in FRP-1 and FRP 2 groups (*p* < 0.05). The uterus, also known as the shell gland, is considered one of the most important parts of the oviduct in chickens. The egg remains here the longest while it is being formed. The uterus has a muscular structure. It has been reported that the egg rotates around its polar axis in the uterus, chalazion forms, and the egg gains a swollen appearance with the penetration of the egg white (45). Since reproductive hormones play an active role in this process, it is thought that the length of the uterus may have increased due to intense muscle movements. In the present study, the lack of impact on follicles and oviducts by the Ferula diet may be related to the dose used and the early laying period of the hens. In a study (39), it was observed that the use of Ferula (100 mg/kg) at lower concentrations in the diets of aged laying hens increased the levels of some reproductive hormones. Hao et al. (39) reported that 100 mg/kg Ferula had higher numbers of small yellow follicles, small white follicles, and middle white follicles. It was also stated in the same study that a 100 mg/kg dose of Ferula increased FSHR, LHR, and ER-*α* activity in the ovary. In a study carried out by Canogullari et al. (36) on Japanese quails, the effects of dietary *F. elaeochytris* (0, 5, and 10 g/kg) root powder supplementation on growth and reproductive performance were investigated. Canogullari et al. (36) reported that Ferula addition in quail feeds increased testicular weight. Additionally, it was also stated that fertility and hatchability rates were totally depressed in Ferula-supplemented groups. In another study (46), the effect of *F. communis* L. root powder (0, 5, and 10%) on reproductive parameters in Awassi ewes was investigated. While the Ferula diets did not affect plasma estrogen concentration or LH peak in their study, the numbers of small and large follicles were found to be significantly influenced. Onal and Güzey (46) determined that the number of small follicles was higher in the ferula-supplemented groups and the number of large follicles was higher in the control group. In another study, Al-Salhie and Al-Hummod (47) examined the effects of *F. hermonis* root extract (0, 100, and 200 mg; orally) on local ducks. Al-Salhie and Al-Hummod (47) determined that serum FSH, LH, and testosterone levels increased in ducks given 200 mg orally. Moreover, the same study revealed that 200 mg Ferula increased seminal tubular diameter and seminiferous epithelium thickness. In a study, it was observed that *F. hermonis* root powder, when used at a dose of 6 mg/kg, showed a strong FSH and LH-like effect in immature rats, as well as moderate estrogenic sexual stimulation (48). Osman (49) stated that *F. hermonis* (3 mg/kg) had a positive effect against testicular damage in male rats induced using *γ*-radiation.

## 5 Conclusion

Ferula is one of the plants exhibiting phytoestrogenic effects. FRP supplementation improved lipid oxidation and increased antioxidant capacity in the present study. Dietary supplementation of FRP decreased serum total cholesterol and triglyceride concentrations. No adverse effects were observed on performance, internal organ weights, and serum enzyme activity. FRP supplementation increased uterus length. Considering the results achieved, it can be seen that FRP does not have a significant estrogenic effect. Even though FRP does not have a significant estrogenic effect, it still can be used to prevent lipid oxidation and for its hypocholesterolemic effect. In conclusion, the lack of expected estrogenic effect of Ferula root on the female reproductive system of laying hens may be associated with the dose added to the diet. Therefore, it is thought to be important to carry out studies at different doses.

## Data Availability

The original contributions presented in the study are included in the article/supplementary material, further inquiries can be directed to the corresponding author.

## References

[1] Mihaylova D Krastanov A Vasilev N. Non-hormonal feed additives as an alternative in animal reproduction. Trakia J Sci. (2020) 18:405–11. doi: 10.15547/tjs.2020.04.016

[2] Ceccarelli I Bioletti L Peparini S Solomita E Ricci C Casini I. Estrogens and phytoestrogens in body functions. Neurosci Biobehav Rev. (2022) 132:648–63. doi: 10.1016/j.neubiorev.2021.12.007 34890602

[3] Sarma HN Sarma I Misra K Saikia A Doima S. Female reproductive health-regulating hormones and phytosteroids as alternative modulator: A review. Annal Multidiscip Res Innov Technol. (2023) 2:45–53.

[4] Dean M Murphy BT Burdette JE. Phytosteroids beyond estrogens: regulators of reproductive and endocrine function in natural products. Mol Cel Endocrinol. (2017) 442:98–105. doi: 10.1016/j.mce.2016.12.013, PMID: 27986590 PMC5276729

[5] Bagheri SM Shieh A Ghalenoei JA Yadegari M Alborzi N. Review of potential spermatogenic and aphrodisiac effects of the Ferula genus. Clin Exp Reprod Med. (2023) 50:143–53. doi: 10.5653/cerm.2023.05995, PMID: 37643827 PMC10477414

[6] Sattar Z Iranshahi M. Phytochemistry and pharmacology of *Ferula hermonis* Boiss.–a review. Drug Res. (2017) 67:437–46. doi: 10.1055/s-0043-109100 28521374

[7] Zanoli P Zavatti M Geminiani E Corsi L Baraldi M. The phytoestrogen ferutinin affects female sexual behavior modulating ERα expression in the hypothalamus. Behav Brain Res. (2009) 199:283–7. doi: 10.1016/j.bbr.2008.12.009, PMID: 19124045

[8] Maiuolo J Musolino V Guarnieri L Macrì R Coppoletta AR Cardamone A. *Ferula communis* L. (Apiaceae) root acetone-water extract: phytochemical analysis, cytotoxicity and in vitro evaluation of estrogenic properties. Plan Theory. (2022) 11:1905. doi: 10.3390/plants11151905, PMID: 35893609 PMC9329896

[9] Sonigra P Meena M. Metabolic profile, bioactivities, and variations in the chemical constituents of essential oils of the Ferula genus (Apiaceae). Front Pharmacol. (2021) 11:608649. doi: 10.3389/fphar.2020.608649, PMID: 33776754 PMC7994278

[10] Salehi M Naghavi MR Bahmankar M. A review of Ferula species: biochemical characteristics, pharmaceutical and industrial applications, and suggestions for biotechnologists. Ind Crop Prod. (2019) 139:111511. doi: 10.1016/j.indcrop.2019.111511

[11] Yang W Chen X Li Y Guo S Wang Z Yu X. Advances in pharmacological activities of terpenoids. Nat Prod Commun. (2020) 18:663–73. doi: 10.1177/1934578X20903555

[12] Rasulev BF Saidkhodzhaev AI Nazrullaev SS Akhmedkhodzhaeva K Khushbaktova ZA Leszczynski J. Molecular modelling and QSAR analysis of the estrogenic activity of terpenoids isolated from Ferula plants. SAR QSAR Environ Res. (2007) 18:663–73. doi: 10.1080/10629360701428631 18038366

[13] Eser N Yoldas A. Identification of heat-resistant chemical components of *Ferula elaeochytris* root extracts by gas chromatographymass spectrometry. Trop J Pharm Res. (2019) 18:55–60. doi: 10.4314/tjpr.v18i1.9

[14] AOAC. Official methods of analysis. 17th Association of Official Analytical Chemists AOAC International Maryland (2000).

[15] Pelicia K Garcia EA Faitarone ABG Silva AP Berto DA Molino AB. Calcium and available phosphorus levels for laying hens in second production cycle. Braz J Poult Sci. (2009) 11:39–49. doi: 10.1590/S1516-635X2009000100007

[16] Vui NV Oanh DH Quyen NTK Linh NT Nang K Phong NH. The impact of adding spirulina algae to drinking water on the productivity, egg quality, yolk lipid oxidation, and blood biochemistry of laying hens. Adv Anim Vet Sci. (2024) 12:1654–63. doi: 10.17582/journal.aavs/2024/12.9.1654.1663

[17] Carvalho CL Andretta I Galli GM Bastos Stefanello T Camargo NDO Mendes RE. Dietary supplementation with β-mannanase and probiotics as a strategy to improve laying hen performance and egg quality. Front Vet Sci. (2023) 10:1229485. doi: 10.3389/fvets.2023.1229485, PMID: 38116507 PMC10728292

[18] Lokapirnasari WP Al-Arif MA Hidayatik N Safiranisa A Arumdani DF Zahirah AI. Effect of probiotics and acidifiers on feed intake, egg mass, production performance, and egg yolk chemical composition in late-laying quails. Vet World. (2024) 17:462–9. doi: 10.14202/vetworld.2024.462-469, PMID: 38595658 PMC11000483

[19] Duman M Şekeroğlu A Yıldırım A Eleroğlu H Camcı Ö. Relation between egg shape index and egg quality characteristics. Eur Poult Sci/Archiv für Geflügelkunde. (2016) 80:1–9. doi: 10.1399/eps.2016.117

[20] Debnath BC Ghosh TK. Phenotypic correlations between some external and internal egg quality traits in Gramapriya layers. Explor Anim. (2015) 5:78–85.

[21] Sarlak S Tabeidian SA Toghyani M Shahraki ADF Goli M Habibian M. Effects of replacing inorganic with organic iron on performance, egg quality, serum and egg yolk lipids, antioxidant status, and iron accumulation in eggs of laying hens. Biol Trace Elem Res. (2021) 199:1986–99. doi: 10.1007/s12011-020-02284-8, PMID: 32666433

[22] Ryu KN No HK Prinyawiwatkul W. Internal quality and shelf life of eggs coated with oils from different sources. J Food Sci. (2011) 76:S325–9. doi: 10.1111/j.1750-3841.2011.02177.x, PMID: 22417448

[23] Carvalho CL Andretta I Galli GM Stefanello TB Camargo NDO Marchiori M. Effects of dietary probiotic supplementation on egg quality during storage. Poultry. (2022) 1:180–92. doi: 10.3390/poultry1030016

[24] Tarladgis BG Pearson AM Dugan LR Jr. The chemistry of the 2-thiobarbituric acid test for the determination of oxidative rancidity in foods. I. Some important side reactions. J Am Oil Chem Soc. (1962) 39:34–9. doi: 10.1007/BF02633347

[25] Lemon DW. An improved TBA test for rancidty new series circular. No:51. Halifax, Nova Scotia: Halifax-Laboratory (1975).

[26] Farıvar A. Düşük ve yüksek deasetilasyon derecesine sahip kitosanın, yumurtacı tavuk rasyonlarında kullanımının verim, kalite ve fonksiyonellik üzerine etkisi. Doktora Tezi Çukurova Üniversitesi, *Fen Bilim Enst*. (2014)

[27] Sudha G Priya MS Shree RI Vadivukkarasi S. In vitro free radical scavenging activity of raw Pepino fruit (*Solanum muricatum* aiton). Int J Curr Pharm Res. (2011) 3:137–40.

[28] Patil AD Dhande PL Bakshi SA Lambate SB. Effects of removal of preen gland on the gross anatomy and biometry of various parts of the oviduct in young and spent hens. J Bombay Vet College. (2014) 21:38–47.

[29] Corp IBM. IBM SPSS statistics for windows, version 21.0. Armonk, NY: IBM Corp (2012).

[30] Faul F Erdfelder E Lang AG Buchner A. G*power 3: A flexible statistical power analysis program for the social, behavioral, and biomedical sciences. Behav Res Methods. (2007) 39:175–91. doi: 10.3758/BF03193146, PMID: 17695343

[31] Duru M Şahı̇n A. Effects of dietary *Ferula eleaochytris* powder on yield and egg quality in laying hens. JIST. (2015) 5:99–108.

[32] Başer KC Özek T Demirci B Kürkçüoǧlu M Aytaç Z Duman H. Composition of the essential oils of *Zosima absinthifolia* (vent.) link and *Ferula elaeochytris* Korovin from Türkiye. Flavour Fragr J. (2000) 15:371–2. doi: 10.1002/1099-1026(200011/12)15:6<371::AID-FFJ919>3.0.CO;2-Z

[33] Duru M. Bazı tıbbi ve aromatik bitki tozlarının farklı taşıyıcılarla kaplanması ile elde edilen yem katkılarının kanatlılarda verim ve metabolizma üzerine etkileri. Doktora Tezi, Mustafa Kemal Üniversitesi. Fen Bilim Enst. (2010):245.

[34] Filik G. Rasyona ilave edilen çakşır (*Ferula eleaochytris*) kökü tozunun yumurtacı tavuklarda yumurta verimi ve kalite özelliklerine etkileri. Yüksek Lisans Tezi, Çukurova Üniversitesi, *Fen Bilim Enst*. (2009):52.

[35] Filik G Bozkurt KA Kutlu HR. Soyasız hazırlanan yumurtacı tavuk yemine östrojenik etkili çakşır (*Ferula eleaochytris*) kökü tozu ilavesinin yumurta verimi ve kalitesine etkileri Ulusal Hayvan Besleme Kongresi (Uluslararası Katılımlı) (2011). 29 p.

[36] Canogullari S Baylan M Copur G Sahin A. Effects of dietary *Ferula elaeochytris* root powder on the growth and reproductive performance of Japanese quail (*Coturnix coturnix japonica*): it is not recommended in a breeder diet. Archiv für Geflügelkunde. (2009) 73:56–60.

[37] Olmez M Şahin T Yılmaz B Riaz R Karadağlu Ö. Effects of dietary supplementation of *Ferula communis* L. on production performance and egg quality parameters of Japanese quail (*Coturnix coturnix japonica*). 2nd international congress of multidisciplinary medical and health sciences studies (2024). 145 p.

[38] Balcı BA Aktop Y. Yeme çakşır otu (*Ferula elaeochytris* K. 1947) ilavesinin japon balığının (*Carassius auratus* L. 1758) büyüme ve gonad gelişimi üzerine etkisi. JIST. (2019) 9:347–59.

[39] Hao E Chang LY Wang DH Chen YF Huang RI Chen H. Dietary supplementation with ferula improves productive performance, serum levels of reproductive hormones, and reproductive gene expression in aged laying hens. *Braz*. J Poult Sci. (2021) 23:eRBCA-2020. doi: 10.1590/1806-9061-2020-1319

[40] Tayeb IT. Effects of replacement *Ferula communis* (Giant fennel) in diet on productive performance and some physiological parameter in Japanese quail. Iraqi J Agric Sci. (2022) 53:1305–11. doi: 10.36103/ijas.v53i6.1645

[41] Khajavi H Hassanabadi A Badouei MA. Effects of adding *Ferula badrakema* root powder with and without sodium saccharin sweetener to the diet on growth performance, immune system and blood metabolites of broiler chickens. Iran J Anim Sci Res. (2023) 15:429–43.

[42] Çopur G Duru M Şahin A Canoğulları S. Çakşır (*Ferula elaeochytris*) kökü tozunun bronz hindilerde yumurta verim ve bazı yumurta verim özelliklerine etkileri. MKU Ziraat Fak Derg. (2004) 9:85–92.

[43] Ghasemi Z Rezaee R Aslani MR Boskabady MH. Anti-inflammatory, anti-oxidant, and immunomodulatory activities of the genus Ferula and their constituents: A review. Iran J Basic Med Sci. (2021) 24:1613–23. doi: 10.22038/IJBMS.2021.59473.13204, PMID: 35432802 PMC8976906

[44] Safari O Sarkheil M Paolucci M. Dietary administration of ferula (*Ferula asafoetida*) powder as a feed additive in diet of koi carp, *Cyprinus carpio* koi: effects on hemato-immunological parameters, mucosal antibacterial activity, digestive enzymes, and growth performance. Fish Physiol Biochem. (2019) 45:1277–88. doi: 10.1007/s10695-019-00674-x, PMID: 31256305

[45] Sönmez M. Reprodüksiyon suni tohumlama ve androloji ders notları. (2012). Fırat Üniv. Veteriner Fak, Turkey, 15–20.

[46] Onal AG Güzey YZ. Effect of *Ferula communis* L. on reproductive parameters in Awassi ewes. J Hellenic Vet Med Soc. (2019) 70:1625–30. doi: 10.12681/jhvms.21785

[47] Al-Salhie KC Al-Hummod SK. *Ferula hermonis* roots extract on testicular biometry and reproductive hormones in local ducks. Indian Vet J. (2019) 96:14–7.

[48] Elmotayam AK Elnattat WM Ali GA El Khatteb RM Abdelhameed MF Ali AH. Assessment of *Ferula hermonis* Boiss fertility effects in immature female rats supported by quantification of ferutinin via HPLC and molecular docking. J Ethnopharmacol. (2022) 289:115062. doi: 10.1016/j.jep.2022.115062 35114339

[49] Osman NN. Antioxidant effects of *Ferula hermonis* and bee honey on γ-radiation-induced oxidative testicular damage in rats. J Radiat Res Appl Sci. (2011) 4:1201–19.

[50] Carpenter KJ Clegg KM. The metabolizable energy of poultry feeding stuffs in relation to their chemical composition. J Sci Food Agric. (1956) 7:45–51. doi: 10.1002/jsfa.2740070109

